# Supplementary material for: Ym1 protein crystals promote type 2 immunity
Source: eLife. 2024 Jan 9;12:RP90676. doi: 10.7554/eLife.90676 (PMC10945506; doi:10.7554/eLife.90676)
Supplement: Supplementary file 1. — Key crystallographic data of ex vivo isolated Ym1 and Ym2 crystals for comparison with in vitro generated recombinant Ym1 and Ym2 crystals. Each dataset was collected from a single crystal. Values in parentheses are for highest-resolution shell. [file elife-90676-supp1.docx]

**Table S1.** **Crystallographic data and refinement statistics**

|  |  | |  | |  | |  | |  |
| --- | --- | --- | --- | --- | --- | --- | --- | --- | --- |
| Protein complex | | Recombinant Ym1 | | *Ex vivo* Ym1 | | Recombinant Ym2 | | *Ex vivo* Ym2 | |
|  | |  | |  | |  | |  | |
| **Data collection** | |  | |  | |  | |  | |
| X-ray source  (beamline) | | SOLEIL  (Proxima 1) | | PETRA III  (P14) | | PETRA III  (P14) | | SLS  (PXI) | |
| Wavelength (Å) | | 0.97857 | | 0.9763 | | 0.9763 | | 1.0000089224 | |
| Space group | | *P21* | | *P21* | | *P21* | | *P21* | |
|  | |  | |  | |  | |  | |
| Cell dimensions | |  | |  | |  | |  | |
| *a*, *b*, *c* (Å) | | 50.33,  60.01,  60.12 | | 50.52,  60.25,  60.12 | | 51.09,  59.90,  59.88 | | 51.26,  60.21,  60.17 | |
|  | |  | |  | |  | |  | |
| α, β, γ (°) | | 90.00,  94.66,  90.00 | | 90.00,  94.48,  90.00 | | 90.00,  94.14,  90.00 | | 90.00,  94.24,  90.00 | |
|  | |  | |  | |  | |  | |
| Resolution (Å) | | 50.00-1.79  (1.90-1.79) | | 59.93-1.42  (1.50-1.42) | | 59.90-1.17  (1.24-1.17) | | 42.50-1.70  (1.81-1.70) | |
|  | |  | |  | |  | |  | |
|  | |  | |  | |  | |  | |
| *R*_meas_ (%) | | 24.7 (112.1) | | 12.2 (138.2) | | 8.0 (35.8) | | 17.7 (136.4) | |
|  | |  | |  | |  | |  | |
| < *I* /σ > | | 5.28 (1.54) | | 8.99 (1.19) | | 15.12 (4.74) | | 6.59 (0.96) | |
|  | |  | |  | |  | |  | |
| CC ½ (%) | | 97.9 (56.6) | | 99.7 (39.5) | | 99.9 (92.5) | | 99.2 (35.8) | |
|  | |  | |  | |  | |  | |
| Completeness (%) | | 99.2 (96.0) | | 98.1 (97.3) | | 98.4 (95.9) | | 97.7 (97.3) | |
|  | |  | |  | |  | |  | |
| Redundancy | | 4.1 (3.9) | | 3.3 (3.3) | | 6.8 (6.1) | | 3.5 (3.4) | |
| ISa score | | 8.04 | | 26.18 | | 27.31 | | 24.20 | |
| Wilson B (Å^2^) | | 20.318 | | 20.936 | | 12.048 | | 24.584 | |
|  | |  | |  | |  | |  | |
| **Refinement** | |  | |  | |  | |  | |
| Resolution (Å) | | 42.40-1.79 | | 26.91-1.42 | | 37.45-1.17 | | 16.78-1.71 | |
|  | |  | |  | |  | |  | |
| No. reflections | | 33 407 | | 66 944 | | 119 181 | | 39 026 | |
|  | |  | |  | |  | |  | |
| *R*_work_ / *R*_free_ (%) | | 17.29 / 19.86 | | 18.10 / 20.52 | | 15.75 / 16.73 | | 17.71 / 20.48 | |
|  | |  | |  | |  | |  | |
| No. non-H atoms | |  | |  | |  | |  | |
| Protein | | 2 957 | | 2 957 | | 2 976 | | 2 970 | |
| Ligand/ion | | 10 | | 4 | | 24 | | 6 | |
| Water | | 215 | | 163 | | 584 | | 239 | |
|  | |  | |  | |  | |  | |
| *B*-factors (Å^2^) | |  | |  | |  | |  | |
| Protein | | 12.09 | | 15.58 | | 10.28 | | 18.50 | |
| Ligand/ion | | 21.52 | | 30.73 | | 16.20 | | 30.69 | |
| Water | | 21.74 | | 22.78 | | 27.02 | | 27.93 | |
|  | |  | |  | |  | |  | |
| R.m.s. deviations | |  | |  | |  | |  | |
| Bond lengths (Å) | | 0.008 | | 0.010 | | 0.010 | | 0.010 | |
| Bond angles (°) | | 0.91 | | 0.98 | | 1.07 | | 0.97 | |
|  | |  | |  | |  | |  | |
